# Asiatic acid protects against cognitive deficits and reductions in cell proliferation and survival in the rat hippocampus caused by 5-fluorouracil chemotherapy

**DOI:** 10.1371/journal.pone.0180650

**Published:** 2017-07-10

**Authors:** Pornthip Chaisawang, Apiwat Sirichoat, Wunnee Chaijaroonkhanarak, Wanassanun Pannangrong, Bungorn Sripanidkulchai, Peter Wigmore, Jariya Umka Welbat

**Affiliations:** 1 Department of Anatomy, Faculty of Medicine, Khon Kaen University, Khon Kaen, Thailand; 2 Center for Research and Development of Herbal Health Products, Khon Kaen University, Khon Kaen, Thailand; 3 School of Life Sciences, Medical School, Queen's Medical Centre, Nottingham University, Nottingham, United Kingdom; 4 Neuroscience Research and Development Group, Khon Kaen University, Khon Kaen, Thailand; Bilkent University, TURKEY

## Abstract

The chemotherapy drug, 5-fluorouracil (5-FU), has been reported to cause cognitive impairments in cancer patients. The drug also reduces cell proliferation and survival in the brain. Asiatic acid (AA) is a triterpene compound found in *Centella asiatica* that can protect against reduction of neurogenesis in the hippocampus and memory deficits induced by valproic acid (VPA). In the present study, we investigated the preventive effects of AA on the deficits in spatial working memory and cell proliferation and survival caused by 5-FU chemotherapy in a rat model. Male Sprague Dawley rats received 5-FU (5 i.v. injections, 25 mg/kg) on day 8, 11, 14, 17 and 20 of the study. This was co-administered with AA (30 mg/kg, oral gavage tube) either 20 days before receiving 5-FU (preventive), after receiving 5-FU (recovery), or for the entire period of the experiment (throughout). Spatial working memory was determined using the novel object location (NOL) test and hippocampal cell proliferation and survival of dividing cells were quantified using immunohistochemistry. Rats in the 5-FU alone and recovery groups showed memory deficits in the NOL test and reductions in cell proliferation and cell survival in the subgranular zone (SGZ) of the hippocampal dentate gyrus. Rats in the control, AA alone, and both preventive and throughout co-administration groups, however, did not exhibit these characteristics. The results showed that 5-FU chemotherapy impaired memory and reduced cell proliferation and cell survival in the SGZ of the hippocampal dentate gyrus. However, these impairments in the animals receiving 5-FU chemotherapy were restored to control levels when AA was co-administered before and during 5-FU treatment. These data demonstrate that AA can prevent the spatial working memory and hippocampal neurogenesis impairments caused by 5-FU chemotherapy.

## Introduction

Chemotherapy drugs are widely used to treat cancer patients, including those suffering from breast cancer. One of its side effects is memory impairment, such as problems with memory formation, attention, and concentration. Memory deficits impact patients quality of life and ability to return to work [1, 2] and have shown to last for many years after chemotherapy treatment [3, 4]. The chemotherapy agent, 5-fluorouracil (5-FU), is commonly used in combination with other agents to treat various cancers [1]. Previous studies have found that 5-FU treatment induces cognitive deficits, including impairments in attention, visual memory, and speed of information processing [5–7]. This drug can cross the blood-brain barrier through simple diffusion and is able to disrupt cell proliferation by blocking the enzyme thymidylate synthase (TS). This enzyme is required for DNA replication [8], which is associated with cell proliferation.

The subgranular zone (SGZ) in the dentate gyrus of the hippocampus is one of two main neurogenic regions where adult neurogenesis takes place and continues throughout life [9–11]. The process of neurogenesis can be influenced by various factors, for example, drugs and environmental stimuli [12, 13]. Furthermore, previous studies have shown that chronic stress decreases neurogenesis [14, 15] and induces apoptosis of new neurons in the hippocampus [16]. The decline in neurogenesis induced by chronic stress can be reversed by the administration of antidepressants [14] Previous investigations have reported that decreases in memory and cell proliferation and survival caused by chemotherapy agents can be mitigated after co-administration with fluoxetine [17]. Moreover, *Kaempferia parviflora* extract can prevent memory deficits and reduction in cell proliferation in the SGZ of the hippocampus [18]. It would be beneficial for cancer sufferers if an agent could be found to ameliorate the side effects of treatment, especially cognitive deficits, which can severely affect these patients quality of life.

Asiatic acid (AA) is a triterpene derived from *Centella asiatica* (L.) Urban. Asiatic acid has been reported to exhibit biological effects, including neuroprotective properties [19–21]. In traditional medicine, *Centella asiatica* has been utilized to restore decreased cognitive function. Several studies have demonstrated that AA enhances learning and memory, which are associated with hippocampal neurogenesis [21–23]. Acute administration of AA (30 mg/kg) has been shown to stimulate learning and memory as measured by a passive avoidance test [24]. In addition, subchronic and chronic administration of AA (30 mg/kg) has been shown to increase cell proliferation in the hippocampus and stimulate spatial working memory [25]. Furthermore, AA (30 mg/kg) can prevent neurogenesis and spatial memory impairment caused by valproic acid (VPA) [26]. Therefore, the present study aims to investigate the biological effects of AA in preventing and restoring cognitive deficit and decreases in hippocampal cell proliferation and survival induced by 5-FU chemotherapy in a rat model.

## Materials and methods

### Animals and drug administration

Adult male Sprague-Dawley rats (weight 180-220g, National Laboratory Animal Center, Mahidol University, Salaya, Nakornpathom) were housed four to five per cage and in a 12 h (7:00 AM to 7:00 PM) light-dark cycle with food and water available *ad libitum*. Animals were weighed daily from arrival and allowed to habituate in the animal facility for one week prior to the start of the procedures. Laboratory animal care in this study was conducted in accordance with the Ethics for Animal Experimentation put forth by the National Research Council of Thailand and with approval from the Animal Ethics Committee of Khon Kaen University under permit number AEKKU 25/2557.

Sixty rats were randomly divided into six groups (n = 10 animals/group). In the first (control) group, rats were administered 0.9% sterile saline 5 times by intravenous (i.v.) injection and received propylene glycol (1 ml/kg/day, Ajax Finechem Pty Ltd., Australia) by oral gavage for 20 days. The second (5-FU) group received 5-FU (25 mg/kg, Boryung pharmaceutical Co., Ltd., Korea) 5 times by i.v. injection on day 8, 11, 14, 17, and 20 of the experiment. Rats in the third (AA) group were given AA (30 mg/kg, dissolved in propylene glycol, Faces Biochemical Co., Ltd., China) administered orally for 20 days. Those in the fourth (preventive) group, animals were administered AA (30 mg/kg) via oral gavage tube for 20 days (from day 1 to day 20) and received 5-FU (5 times by i.v. injection) at an equivocal dose as the rats in group two. The fifth (recovery) group, animals were administered 5-FU (5 times by i.v. injection) at an equivocal dose as the rats in group two and received AA 1 day after the last 5-FU injection (from day 21 to day 40) for 20 days of the experiment. Rats in the sixth (throughout) group, animals were administered 5-FU (5 times by i.v. injection) at an equivocal dose as the rats in group two and received AA for the entire period of the experiment (from day 1 to day 40), a total of 40 days. Animals in all groups were given 3 BrdU injections (100 mg/kg at a volume of 4 ml/kg, Sigma Aldrich, Inc., St. Louis, USA) by intraperitoneal (i.p.) injection. These BrdU injections were administered 24 hours apart beginning 2 days before their 5-FU/saline injection.

### Behavioral testing

#### Novel object location (NOL)

The NOL test was used to determine spatial working memory in the present study. The protocol was adapted from a previous method [27] and carried out as previously described [25, 28]. The task apparatus consisted of an arena (36 cm x 50 cm x 30 cm) and plastic bottles filled with water to weigh them down. The experiment was conducted at an illumination of 350–400 lux and recorded by VDO camcorder Version-052, OKER (Crown computer Co., Ltd, Bangkok, Thailand). One day prior to testing, each animal was habituated to an arena for 30 min.

The NOL test began 3 days after the end of the drug treatment and was composed of two-trials (familiarization and choice trials). In the familiarization trial, two identical objects were randomly placed in separate locations in the arena and the rats were allowed to explore the objects for 3 min. Then, the rats were returned to their home cages for a 15 min inter-trial interval. Meanwhile the objects and the arenas were cleaned with 20% ethanol to eliminate olfactory cues. During the choice trial, one object was returned to the same location while the other one was moved to a new (novel) location, after which the rats were returned to the arena and allowed to explore the objects for 3 min. The exploratory activity that was measured was time spent actively exploring the object by sniffing, licking, chewing, or directing the nose towards the object at a distance less than 2 cm [27]. The preference index (PI) was defined as the percentage of exploration time spent examining the object in the novel location over the total exploration time (spent with objects in both novel and familiar locations) during the choice trial as described by Sirichoat et al. (2015) [25] and Umka Welbat et al. (2016) [26].

### Tissue preparation

After the behavioral test, rats were euthanized by rapid stunning and cervical dislocation. Brains from each group were cryoprotected in a 30% sucrose solution for 3 hours at 4^°^C and then embedded in Optimal Cutting Temperature (OCT) compound (Thermo Fisher Scientific, Germany). They were then snap-frozen in liquid nitrogen-cooled isopentane and stored at −80^°^C prior to sectioning.

### Immunohistochemistry

Ki-67 was used to quantify cell proliferation in the dentate gyrus of the hippocampus. Frozen brains were serially sectioned (20 μm) in the coronal plane from the Bregma point -2.3 to -6.3 mm to include the entire dentate gyrus using a cryostat. Sections were mounted on 3-aminopropyl-methoxysilane (APS)-coated slides and stored at −20^°^C for Ki-67 staining. Every 15^th^ section from the length of the dentate gyrus was used to select a total of 9 sections per brain using a systemic random sampling technique [29]. Ki-67 staining was performed as previously described [28]. Sections were incubated with monoclonal mouse anti Ki-67 primary antibody (1:150, Vector Laboratory, Inc., USA.) for 1 h. Sections were then incubated with 488 Rabbit Anti-mouse IgG (1:300,Invitrogen, USA) for 40 min and counter-stained with propidium iodide (1:6000, Sigma Aldrich, Inc., St. Louis, MO, USA) for 30 sec.

Cell survival was investigated using BrdU immunostaining. Frozen brains were serially sectioned (40 μm) in the coronal plane from the Bregma point -2.3 to -6.3 mm to include the entire dentate gyrus using a freezing microtome. Sections were kept in a cryoprotective buffer at 4^°^C. Every 8^th^ section from the length of dentate gyrus was used to select a total of 9 sections per brain. Sections were then incubated with sheep primary anti-BrdU (1:100, Abcam, UK) at 4^°^C overnight. Following this, they were incubated with anti-sheep secondary Alexa Fluor 488 (1:300, Invitrogen, USA) for 1 h and counter-stained with propidium iodide (1:6000, Sigma Aldrich, Inc., St. Louis, MO, USA) for 30 sec.

All sections were viewed at X40 on a Nikon ECLIPSE 80i fluorescence microscope running NIS-Element AR 3.2 software (Melville, NY, USA). Then Ki-67 rand BrdU positive cells within the SGZ (defined as 3 cell diameters from inside of the dentate gyrus) were counted for both blades of the dentate gyrus [1, 17, 30]. The numbers of Ki-67 and BrdU positive cells in each hippocampus were produced by combining the cell counts per section for the entire dentate gyrus and multiplying the results by 15 and 8, respectively.

### Statistical analysis

All statistical parameters were calculated using GraphPad Prism (Ver. 5.0) software, and expressed as mean±standard error of mean (SEM). A probability level of *p*<0.05 was considered statistically significant. Data were analyzed via a paired Student’s *t*-test and an ANOVA (analysis of variance) test when appropriate. All parameters were performed blind.

## Results

### Effect of 5-FU and AA on spatial working memory

**Fig 1:**
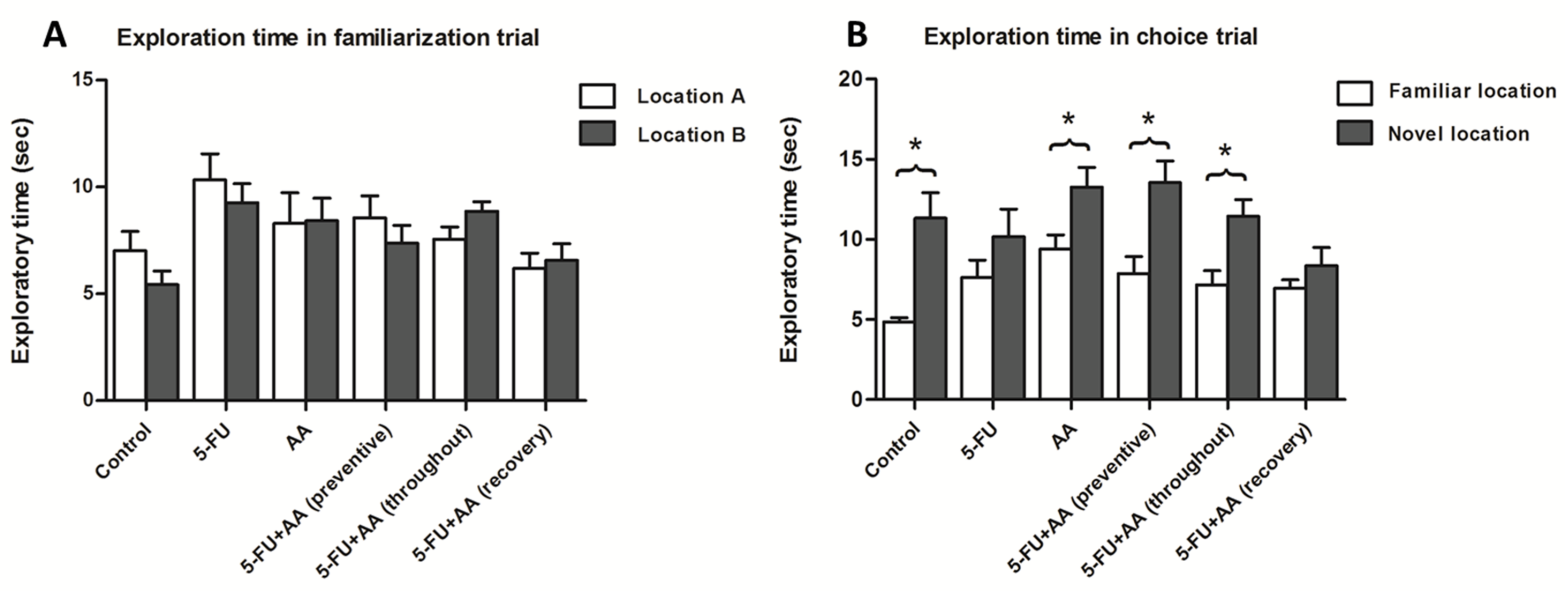
**Mean exploration times (mean ± SEM) of animals during the familiarization (A) and choice (B) trials of the NOL test after treatment.** In the familiarization trial, animals from all groups showed no significant difference in the total exploration time of the two objects (*p*>0.05). In the choice trial, the control, AA, 5-FU+AA (preventive), and 5-FU+AA (throughout) groups spent significantly longer exploring the object in the novel location compared with that in the familiar location (*p<0.05), whereas the 5-FU and 5-FU+AA (recovery) groups did not (*p*>0.05).

The behavioural effect of 5-FU and AA on spatial working memory was determined using the NOL test three days after the end of the drug administration. During the familiarization trial, the animals explored two identical objects. There were no significant differences among the six groups regarding to exploration time for either object in the arena (mean±SEM; location A, control: 7.013±0.907 sec, 5-FU: 10.33±1.226 sec, AA: 8.296±1.437 sec, preventive: 8.551±1.024 sec, throughout: 7.557±0.578 sec, recovery: 6.196±0.710 sec.; location B, control: 5.433±0.631 sec, 5-FU: 9.263±0.897 sec, AA: 8.413±1.071 sec, preventive: 7.387±0.823 sec, throughout: 8.873±0.433 sec, recovery: 6.570±0.772 sec., *p*>0.05, paired Student *t*-test, Fig 1A), indicating that the animals did not display a preference for either object’s location over the other. In the choice trial, one object was moved to a novel location and animals were tested for their ability to discriminate between the objects placed in the familiar and novel locations. Animals in the control, AA, 5-FU+AA (preventive), and 5-FU+AA (throughout) groups spent a significantly longer time exploring the object in the novel location compared to the object in the familiar location (mean±SEM; familiar location, control: 4.839±0.268 sec, AA: 9.390±0.877 sec, preventive: 7.859±1.072 sec, throughout: 7.164±0.883 sec.; novel location, control: 11.320±1.593 sec, AA: 13.260±1.235 sec, preventive: 13.540±1.345 sec, throughout: 11.440±1.039 sec., *p*<0.05, paired Student *t*-test, Fig 1B), indicating unaffected spatial working memory. In contrast, animals treated with 5-FU alone and 5-FU with AA (recovery) could not discriminate between the object placed in the novel location and that in the familiar location (mean±SEM; familiar location, 5-FU: 7.627±1.088 sec, recovery: 6.951±0.530 sec.; novel location, 5-FU: 10.160±1.736 sec, recovery: 8.363±1.121 sec., *p*>0.05, paired Student *t*-test), suggesting that 5-FU induced a deficit in spatial working memory.

**Fig 2:**
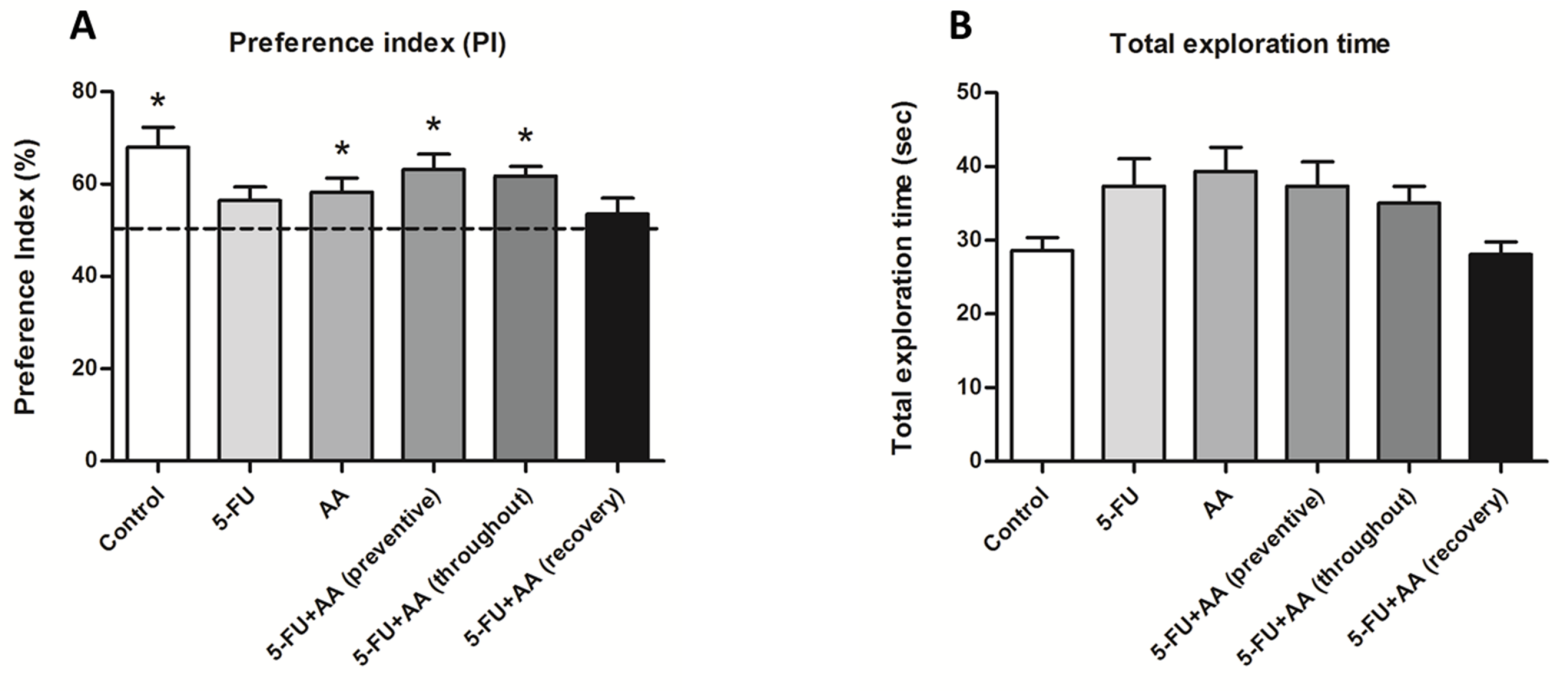
Exploration times of animals in NOL test. The preference index (PI) were significantly higher than 50% (chance) in the control, AA, 5-FU+AA (preventive), and 5-FU+AA (throughout) groups (*p<0.05, **A**), whereas the PI in the 5-FU and 5-FU+AA (recovery) groups did not deviate significantly from chance (*p*>0.05). Total exploration time of the familiarization and choice trials (**B**) did not differ significantly among the various groups (*p*>0.05).

The exploration times in the choice trial were converted into a preference index (PI). The mean PI of the control, AA, 5-FU+AA (preventive), and 5-FU+AA (throughout) groups was significantly higher than 50% chance (mean±SEM; control: 67.95±4.37, AA: 58.21±3.06, preventive: 63.16±3.32, throughout: 61.78±2.04., *p*<0.05; one-sample *t*-test, Fig 2A), suggesting that these animals did not exhibit memory deficit. In contrast, the PI of the 5-FU and 5-FU+AA (recovery) groups did not differ from 50% chance, indicating memory impairment (mean±SEM; 5-FU: 56.41±2.99, recovery: 53.52±3.45., *p*>0.05; one-sample *t*-test). Moreover, there was no significant difference among the groups in terms of total exploration time (mean±SEM; control: 28.60±1.74, 5-FU: 37.37±3.68, AA: 39.35±3.24, preventive: 37.33±3.30, throughout: 35.03±2.30, recovery: 28.07±1.70., *p*>0.05, one-way ANOVA, Bonferroni’s post-hoc test, Fig 2B), indicating that animals did not suffer from impairment of locomotor activity.

### Effect of 5-FU and AA on proliferating cell counts in the dentate gyrus

**Fig 3:**
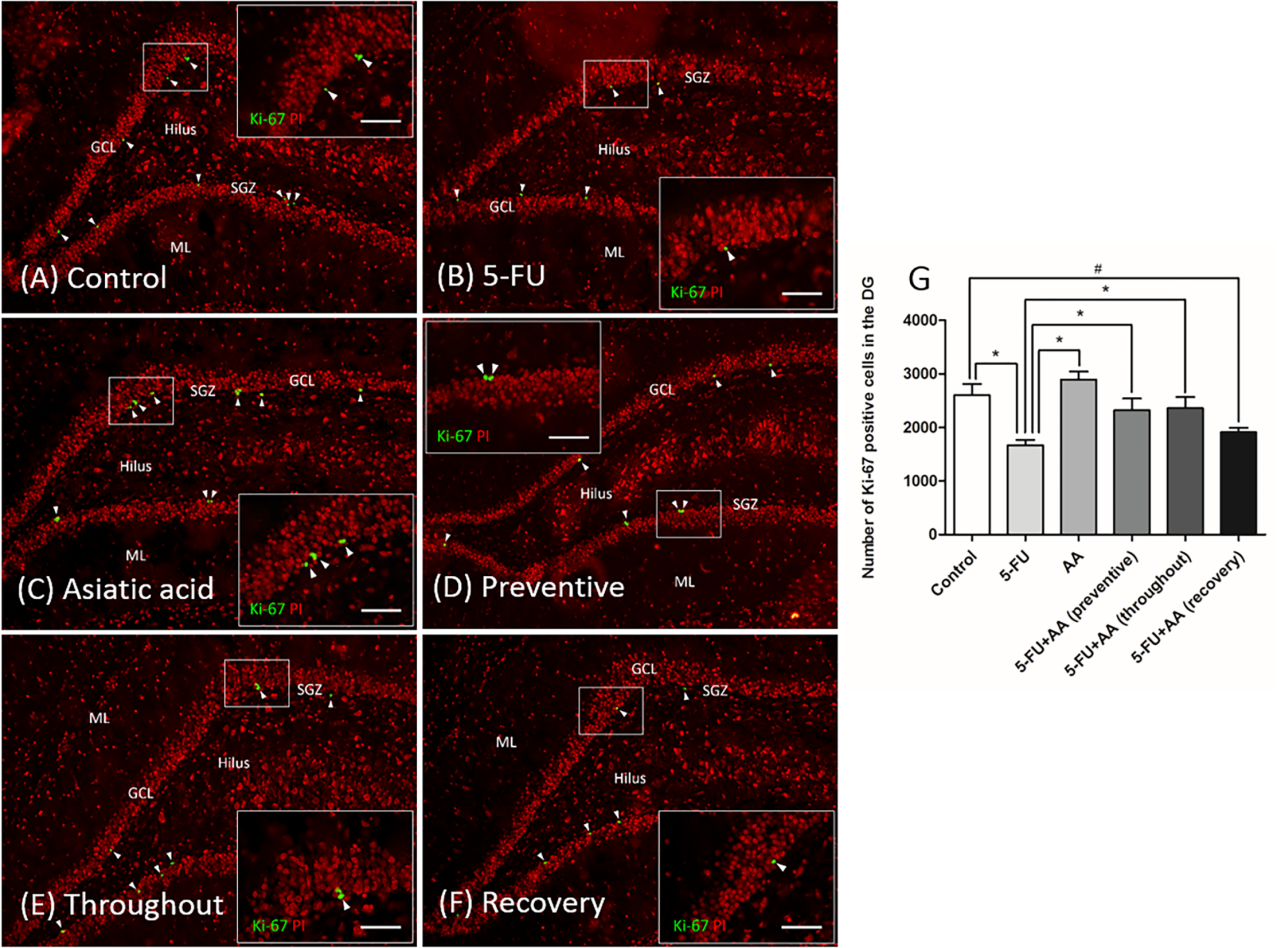
Effect of 5-FU and AA on proliferating cell counts in the dentate gyrus. Representative images of Ki-67 positive cells in the dentate gyrus in each group **(A-F)**. Ki-67 positive cells were stained green in the subgranular zone (SGZ) of the dentate gyrus. Section were counterstained with red nuclear dye, propidium iodide: PI. Arrowheads indicate Ki-67 positive cells in the the dentate gyrus (scale bars: 100 μm). Inserted images show Ki-67 immunostaining under high magnification (scale bar: 50 μm). Mean Ki-67 positive cell counts of the 5-FU group were significantly lower than the control, AA, and 5-FU+AA (preventive and throughout) groups (*p<0.05, **G**). Inaddition, Ki-67 positive cell number in the 5-FU+AA (recovery) group were significantly lower than in the control group (#*p*<0.05, **G**). ML: molecular layer, GCL: granule cell layer.

Fig 3A–3F show representative images of proliferating cells within the SGZ of the dentate gyrus obtained using Ki-67 immunohistochemistry. The number of Ki-67 positive cells differed significantly among the groups. Animals receiving 5-FU alone showed a significant decrease in Ki-67 positive cells compared with the controls (mean±SEM; control: 2605±84.02 cells, 5-FU: 1668±41.25 cells, *p*<0.05, one-way ANOVA, Bonferroni’s post-hoc test, Fig 3G). The Ki-67 positive cell numbers in the group treated with AA on its own did not differ significantly compared to the control (mean±SEM; AA: 2895±61.36 cells, p>0.05, one-way ANOVA, Bonferroni’s post-hoc test), but 5-FU group (*p*<0.05, one-way ANOVA, Bonferroni’s post-hoc test). The Ki-67 positive cell numbers of the groups in which 5-FU and AA were co-administered (preventive and throughout) did not differ from the controls (mean±SEM; preventive: 2320±90.80 cells, throughout: 2360±85.18 cells, *p*>0.05, one-way ANOVA, Bonferroni’s post-hoc test), but were significantly higher than in the 5-FU group (*p*<0.05, one-way ANOVA, Bonferroni’s post-hoc test). However, subjects in the 5-FU and AA (recovery) group had a significantly lower numbers of Ki-67 positive cells compared with controls (mean±SEM; recovery: 1913±33.49 cells, *p*<0.05, one-way ANOVA, Bonferroni’s post-hoc test). These results indicate that 5-FU induced a reduction of cell proliferation in the SGZ of the dentate gyrus. This effect can be prevented by AA if it is administered before or during the 5-FU treatment period, but not after.

### Effect of 5-FU and AA on cell survival in the dentate gyrus

**Fig 4:**
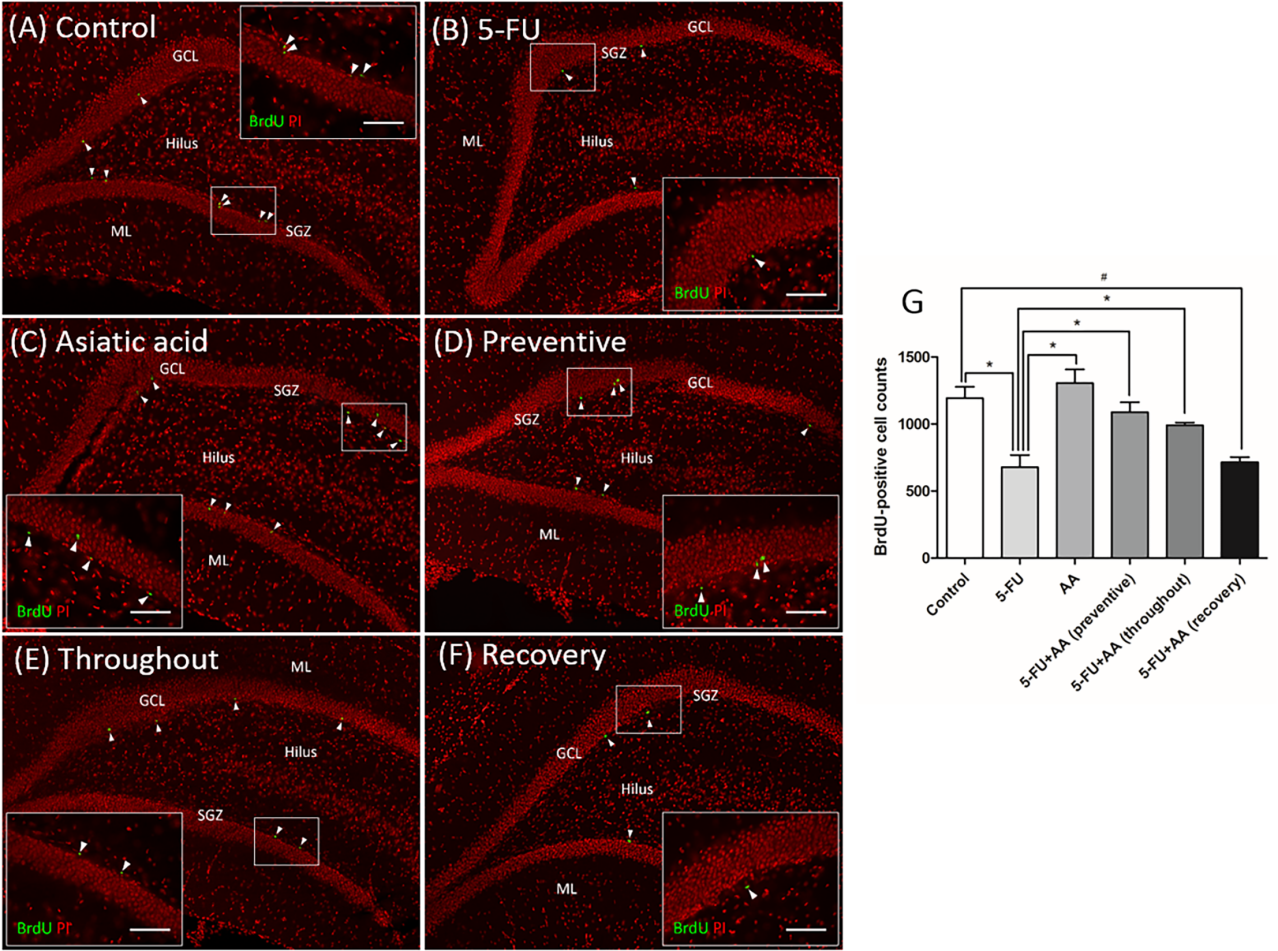
Effect of 5-FU and AA on cell survival in the dentate gyrus. Representative images of BrdU positive cells in the dentate gyrus in each group **(A-F)**. BrdU positive cells were stained green in the subgranular zone (SGZ) of the dentate gyrus. Sections were counterstained with red nuclear dye, propidium iodide: PI. Arrowheads indicate BrdU positive cells in the the dentate gyrus (scale bars: 100 μm). Inserted images show BrdU immunostaining under high magnification (scale bar: 50 μm). Mean BrdU positive cell counts in the 5-FU group were significantly lower than the control, AA, and 5-FU+AA (preventive and throughout) groups (*p<0.05, **G**). Furthermore, BrdU positive cell counts in the 5-FU+AA (recovery) group were significantly less than those in the control group (#*p*<0.05, **G**). ML: molecular layer, GCL: granule cell layer.

In order to study cell survival, BrdU injections were administered daily for three days prior to 5-FU treatment. BrdU positive cells were counted in the SGZ of the dentate gyrus (Fig 4A–4F). There was a significant decrease of BrdU positive cells in animals treated with 5-FU alone compared with controls (mean±SEM; control: 1193±85.62 cells, 5-FU: 677.5±92.34 cells, *p*<0.05, one-way ANOVA, Bonferroni’s post-hoc test, Fig 4G). Animals receiving AA alone had significantly higher numbers of BrdU positive cell compared to the 5-FU group (mean±SEM; AA: 1305±103.4 cells, *p*<0.05, one-way ANOVA, Bonferroni’s post-hoc test). The BrdU-positive cell numbers in two of the groups treated with both 5-FU and AA (preventive and throughout) did not differ significantly from the control group (mean±SEM; preventive: 1088±75.37 cells, throughout: 990.0±21.56 cells, *p*>0.05, one-way ANOVA, Bonferroni’s post-hoc test), but were significantly higher than those in the 5-FU group (*p*<0.05, one-way ANOVA, Bonferroni’s post-hoc test). In contrast, animals treated with both 5-FU and AA (recovery) had significantly fewer BrdU positive cells when compared with controls (mean±SEM; recovery: 715.0±39.62 cells, *p*<0.05, one-way ANOVA, Bonferroni’s post-hoc test). These results demonstrated that AA can protect against decreased cell survival when administered before or during 5-FU treatment, but not after.

## Discussion

The data presented shows that treatment with 5-FU chemotherapy caused cognitive deficits, which were associated with decreases in the rate of neurogenesis in the SGZ of the dentate gyrus. These impairments were mitigated through co-administration of 5-FU with AA before (preventive) and during (throughout) but not after (recovery) 5-FU treatment.

Several studies have indicated that 5-FU administered at a high single dose [31–33] or repeated doses [1, 30, 34] can produce cognitive impairment. These effects can persist for several years after treatment [3, 4, 6, 35–37] and the cause is still unclear [38]. The NOL test was chosen as a test of spatial working memory as it is a hippocampal-dependent task. This test relies on the spontaneous preference of rats to explore an object location without requiring positive or negative reinforcements [27, 39]. Our results showed that control animals were able to discriminate between objects in novel and familiar locations. In contrast, animals which had received 5-FU showed no significant difference in the time spent on the two objects, indicating a deficit in spatial working memory. These results are consistent with earlier reports that treatment with 5-FU induces cognitive impairment in both animals [30, 34, 40] and humans [41, 42]. Although the preference index of the AA group did not differ from control group, animals administered with AA alone showed an improvement in their ability to distinguish between objects placed in familiar and novel locations. This suggests that treatment with AA alone for 20 days did improve spatial working memory. Previous studies have found that treatment with AA alone for 28 days show preference index higher than control, indicating that AA alone can enhance spatial working memory when compared with control [25]. However, in the present study, the preference index in animals receiving only AA was not different from control. It might be due to a shorter period (20 days) of AA administration. In addition, AA has cognitive benefits for animals treated with VPA when administered prior to VPA treatment [26]. Animals receiving AA before and during 5-FU treatment showed a significant preference for the object in the novel location compared with that in the familiar location. However, animals receiving 5-FU alone or that were co-treated with AA after 5-FU treatment did not differentiate between the objects in the novel and familiar locations. This suggests that AA provides cognitive benefits and can protect against the some of the negative effects of this chemotherapy agent, but only if administered before or during (not after) chemotherapy treatment.

New neurons are continuously produced throughout life from a population of dividing cells within the adult rat brain [43]. New neurons in the hippocampus have the ability to proliferate, differentiate and migrate into the SGZ of the hippocampal dentate gyrus in order to connect into the brain’s neural network [44]. Moreover, hippocampal neurogenesis is associated with learning and memory [45, 46]. The production of new neurons in the SGZ of the dentate gyrus is important for spatial memory formation [47, 48]. Reductions in cell proliferation and survival in the SGZ are influenced by various factors such as environmental and pharmacological stimuli [44, 49], irradiation [50, 51], or chemotherapy drugs [2, 17, 34, 52]. The main function of 5-FU is to inhibit thymidylate synthase, which is required for thymidine synthesis during the S-phase of the cell cycle [53, 54]. In the present study, the effects of 5-FU and AA on production and survival of new hippocampal cells were examined using immunohistochemistry to analyze Ki-67 and BrdU, respectively. The results showed that the number of Ki-67 and BrdU positive cells in 5-FU-treated animals was significantly lower than in the control animals. These results confirmed previous reports that 5-FU treatment reduced cell proliferation and cell survival in the SGZ of the dentate gyrus [1, 2]. The animals receiving AA after 5-FU treatment (recovery) showed the same reduction in both the cell proliferation and cell survival as the group treated 5-FU alone. While AA on its own had no effect on cell proliferation and cell survival, co-administration of AA before (preventive) and during (throughout) 5-FU treatment led to there being no reduction in cell proliferation or cell survival after the end of the experiment when compared to controls. This indicates that AA is able to prevent the cognitive deficits associated with a reduction of hippocampal neurogenesis, but that these deficits cannot be reversed after 5-FU chemotherapy treatment.

Previous reports have demonstrated that AA has antioxidant and neuroprotective activities [55] which have been shown to protect against neuronal damage in cell cultures [22]. Similarly, AA has been shown to protect against mitochondria injury from cerebral ischemia in animal models [21]. A recent study has found that AA can also prevent spatial working memory and hippocampal neurogenesis impairment caused by VPA [26]. Our results show that co-administration of AA and 5-FU ameliorated the reduction in cell proliferation and survival in the SGZ and improved memory as measured by the NOL test. This change may be due to the neuroprotective and antioxidant properties of AA.

The extract of *Centella asiatica* can increase levels of brain-derived neurotrophic factor (BDNF) [56], which is associated with increasing the proliferation of neural stem cells [57]. A previous study showed that AA, a compound extracted from *Centella asiatica*, can increase levels of doublecortin (DCX) and Notch1 protein expression within the hippocampus [25] DCX is a microtubule-associated protein that is highly expressed in immature neurons [58, 59]. It is also required for neuronal migration, differentiation, and plasticity of the cerebral cortex [58, 60] Notch1 plays an important role in the process of neurogenesis and is also expressed in the dendrites of mature neurons [61, 62, 63]. Therefore, the enhancement of cell proliferation and survival and spatial working memory by asiatic acid in this study may be via the mechanism of BDNF, DCX and Notch1 protein expression.

In conclusion, the results of this study show that co-administration of AA can counteract the memory impairment associated with a reduction in new neuron generation in the SGZ of the hippocampus caused by 5-FU. Our results provide evidence that AA might be useful in preventing cognitive impairment in patients treated with chemotherapy drugs. Investigations of other mechanisms of AA on neurogenesis and its interaction with chemotherapy agents will help doctors better understand how to counteract memory deficits in patients undergoing these treatments.
